# The COVIDTW3 Study: Impact of Variants of Concern and Vaccination on Mortality in Intubated Patients with COVID-19-Related Respiratory Failure from 2021 to 2023

**DOI:** 10.3390/biomedicines14040756

**Published:** 2026-03-26

**Authors:** Kuan-Chun Wong, I-Shiang Tzeng, Tsung-Han Hsieh, Chan-Yen Kuo, Chih-Wei Wu

**Affiliations:** 1 Department of Pharmacy, Taipei Tzu Chi Hospital, Buddhist Tzu Chi Medical Foundation, New Taipei City 231016, Taiwan; owenwong1994@gmail.com; 2 Department of Research, Taipei Tzu Chi Hospital, Buddhist Tzu Chi Medical Foundation, New Taipei City 231016, Taiwan; istzeng@gmail.com (I.-S.T.); b87404037@gmail.com (T.-H.H.); cykuo863135@gmail.com (C.-Y.K.); 3 Division of Pulmonary Medicine, Department of Internal Medicine, Taipei Tzu Chi Hospital, Buddhist Tzu Chi Medical Foundation, New Taipei City 231016, Taiwan

**Keywords:** COVID-19, Alpha variant, Omicron variant, vaccination, body mass index, invasive mechanical ventilation, mortality

## Abstract

**Background:** In recent years, the severity of COVID-19 has diminished. However, some patients progressed to respiratory failure, necessitating intubation and mechanical ventilation. This study investigated the impact of variants of concern and vaccination status on mortality in mechanically ventilated patients. **Method:** We conducted a retrospective analysis of the medical records of intubated COVID-19 patients from 1 January 2021, to 31 December 2023. Patients who received at least one dose of a vaccine were classified as vaccinated, and variant types were classified based on the dominant variant reported by the Taiwan Centers for Disease Control. The primary outcome measured was time from intubation to all-cause in-hospital death. **Result:** A total of 254 patients were analyzed, comprising 65 patients infected with the Alpha variant and 189 with the Omicron variant. Clinical data, including variant type, vaccination status, and SOFA score at the time of intubation, were meticulously recorded. The overall mortality rate was 40%, with two epidemic surges occurring in 2021 and 2022. Infection with the Alpha variant was associated with a significantly higher risk of mortality (adjusted hazard ratio = 5.42 (2.78–10.7); *p* < 0.01). Key prognostic factors identified included age, body mass index, SOFA score, and serum bicarbonate levels. **Conclusions:** The overall mortality rate remained notably high. The study identified several factors associated with increased mortality risk, including older age, higher SOFA scores, Alpha variant infection, decreased serum bicarbonate levels, and lower BMI. However, vaccination status was not a significant prognostic indicator.

## 1. Introduction

In December 2019, a novel virus known as severe acute respiratory syndrome coronavirus 2 (SARS-CoV-2) triggered the COVID-19 pandemic. The virus first emerged in China and subsequently spread rapidly across the globe. At the onset of the pandemic, Taiwan effectively managed to control the outbreak. Several factors contributed to this successful epidemic prevention, including the island’s geography, stringent border controls, and rigorous quarantine measures. Taiwan has a population of approximately 23 million. In 2020, the total number of confirmed COVID-19 cases was 823, according to open data from the Taiwan Centers for Disease Control (CDC) [1]. Due to successful anti-epidemic measures implemented in 2020, Taiwan experienced a one-year delay in epidemic onset compared to other countries. In 2021, Taiwan faced a small-scale outbreak that resulted in approximately 16,000 confirmed cases, followed by a large-scale outbreak in 2022 that led to around 9 million confirmed cases [2]. This unique epidemic pattern in Taiwan resulted in a single predominant variant each year. Genomic sequencing identified the variant of concern (VOC) as the Alpha variant (B.1.1.7) in 2021 [3,4] and the Omicron variant (B.1.1.529) in 2022 and 2023 [4,5].

Although the Omicron variant exhibited increased infectivity compared to previous VOCs, the severity of COVID-19 decreased substantially [6]. However, the mortality rate among intubated patients with COVID-19-related acute respiratory distress syndrome (ARDS) remains high. A study conducted in the United States during the first half of 2020 found that the mortality rate for intubated patients with COVID-19-related ARDS was 30% [7]. The COVIDTW study reported a 45% mortality rate among intubated patients infected with the Alpha variant in Northern Taiwan in 2021 [8]. As the severity of COVID-19 has decreased over time, few studies have specifically focused on the prognosis of intubated patients with ARDS related to the Omicron variant [9,10,11]. A single-center study conducted in Japan from 2020 to 2023 revealed a 31% mortality rate among intubated patients infected with the Omicron variant [9]. In addition, the COVIDTW2 study reported a 39% mortality rate associated with the Omicron variant in Taiwan in 2022 [10].

Several risk factors for mortality have been identified in the literature. Adverse prognostic factors include advanced age, multiple comorbidities, and high Sequential Organ Failure Assessment (SOFA) scores, among others [8,9,10,11,12,13,14,15,16]. Elderly patients are disproportionately affected by severe COVID-19, facing higher risks of hospitalization and respiratory failure. These individuals are also more likely to present with multiple comorbidities and experience more rapid waning of vaccine-induced immunity, necessitating booster doses to maintain adequate protection against severe disease and death [17]. The Omicron variant has reduced the overall disease severity. However, several studies have shown that different viral variants did not have a significant effect on the survival of intubated patients [9,11,12]. Although vaccination reduced the risk of disease progression to critical illness, the role of vaccination in intubated patients with respiratory failure due to COVID-19 remains unclear [9,10,13]. In this study, we specifically focused on intubated patients with COVID-19-related ARDS. Our aim was to compare the severity of the disease between the Alpha and Omicron variants and to analyze prognostic factors, including vaccination status.

## 2. Materials and Methods

We conducted a retrospective review of the medical records of patients with COVID-19 at Taipei Tzu Chi Hospital from 1 January 2021 to 31 December 2023. Clinical data were accessed for research purposes on 12 January 2024. SARS-CoV-2 infection was diagnosed using a reverse-transcription polymerase chain reaction assay or an antigen test performed on a respiratory specimen. We specifically included patients who required invasive mechanical ventilation (IMV) due to COVID-19-related ARDS. Patients younger than 18 years, those with incomplete medical information, or individuals who were intubated for surgical procedures were excluded from the study. Genomic sequencing of viral variants was not performed in this research. The predominant VOC was the Alpha variant in 2021 and the Omicron variant in 2022 and 2023, as reported in previous studies [2,3,4,5]. This study is an extension of our earlier work (COVIDTW [8] and COVIDTW2 [10]), and we have designated it as the COVIDTW3 study. Day 1 was defined as the date of intubation for invasive mechanical ventilation.

We reviewed the electronic medical records of intubated patients with COVID-19-related ARDS and retrieved data including age, sex, body mass index (BMI), vaccination status, smoking history, bedridden status, education level, marital status, out-of-hospital cardiac arrest prior to admission, comorbidities, the SOFA score on day 1, arterial blood gas (ABG) data on day 1, and specific medications administered for COVID-19. Education level was categorized into two groups based on the attainment of a bachelor’s degree. Mental disorders included bipolar disorder, depression, and schizophrenia. Autoimmune diseases encompassed systemic lupus erythematosus, Sjögren’s syndrome, and rheumatoid arthritis. Neuromuscular diseases included myasthenia gravis, muscular dystrophy, Kennedy’s disease, and neuronal intranuclear inclusion disease. The oral antiviral medications administered included nirmatrelvir/ritonavir (Paxlovid) and molnupiravir (Lagevrio). According to the electronic medical records, no cases of COVID-19 reinfection or influenza coinfection were documented among our patients.

Using an in-hospital medical network system, vaccination status could be queried from the Taiwan CDC. In the COVIDTW3 study, patients who received at least one dose of a COVID-19 vaccine were classified as vaccinated, while those without vaccination were classified as unvaccinated. The COVIDTW3 study included four types of COVID-19 vaccines: MVC-COV1901 (Medigen vaccine), BNT162b2 (Pfizer-BioNTech vaccine), mRNA-1273 (Moderna vaccine), and ChAdOx1 nCoV-19/AZD1222 (AstraZeneca). Individuals received various vaccine combinations according to the guidelines set forth by the Taiwan CDC. For elderly patients, a booster dose was recommended to achieve sufficient levels of neutralizing antibodies. During the COVIDTW3 study period (2021–2023), heterogeneous vaccine platforms were considered the most effective combinations for booster doses [18]. We divided the cohort into three groups: Group 1, patients who received a heterogeneous booster; Group 2, patients without vaccination; and Group 3, the remaining subjects, excluding those in Groups 1 and 2. Patients in Group 3 had received at least one vaccine dose but exhibited complex vaccination combinations. We analyzed the effects of different vaccine types by comparing the mortality among these three groups.

The primary outcome was the time from intubation to all-cause in-hospital mortality. The secondary outcomes included the length of hospital stay, duration of IMV, occurrences of septic shock, septicemia, cytomegalovirus (CMV) infection, Aspergillus infection, utilization of extracorporeal membrane oxygenation (ECMO), in-hospital cardiac arrest, in-hospital stroke, in-hospital seizure, acute kidney injury, empyema, pneumothorax, and post-tracheostomy status. Septicemia encompassed both bacteremia and fungemia. CMV infection was defined as a serum CMV viral load of ≥500 IU/mL or a positive CMV-IgM test. Aspergillus infection was characterized by a positive sputum culture or an Aspergillus galactomannan antigen level of ≥0.5 (optical density index) in serum or bronchoalveolar lavage fluid. Acute kidney injury was defined as an increase in serum creatinine to ≥1.5 times baseline during hospitalization. Post-tracheostomy status indicated that the patient had undergone tracheostomy surgery after admission.

Numerical data are presented as medians with interquartile ranges (IQRs), while categorical data are presented as counts and percentages. The Mann–Whitney U test was employed to compare differences in medians for numerical data. For categorical data, Pearson’s chi-square test or Fisher’s exact test was utilized to assess differences in proportions. The Cox proportional hazards model was applied to identify significant risk factors influencing survival rates. The Bonferroni correction was employed to adjust *p*-values for multiple comparisons. We determined the optimal cut-off points for mortality risk factors using the receiver operating characteristic (ROC) curve and the Youden index. Statistical significance was defined as a *p*-value of less than 0.05. The statistical analyses were conducted using GraphPad Prism (version 10.3.1). The R software (version 4.4.2) was utilized for the restricted cubic spline analysis.

The Institutional Review Board of Taipei Tzu Chi Hospital, Buddhist Tzu Chi Medical Foundation, approved the study (protocol number: 13-IRB021, approved on 21 February 2024) and waived the requirement for informed consent. Access to identifying information was restricted to the corresponding author. Other authors only had access to de-identified data for analysis purposes.

## 3. Results

**Figure 1:**
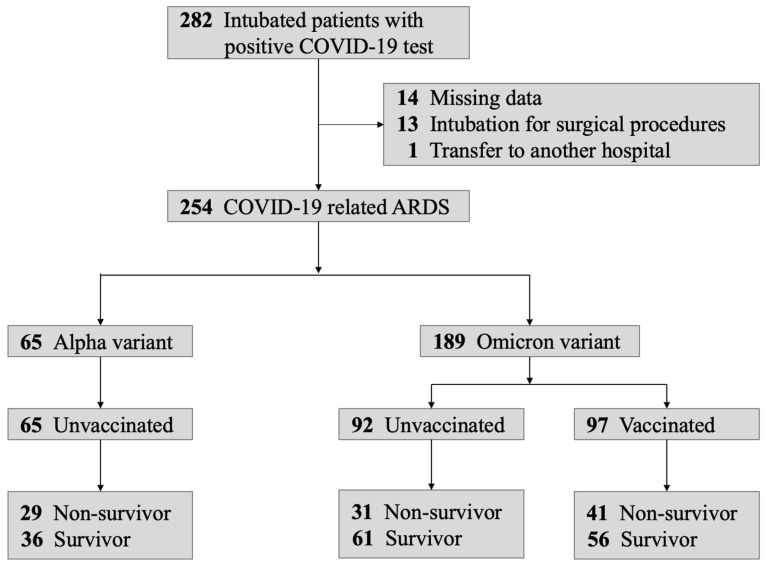
Patient enrollment flow diagram.

**Figure 2:**
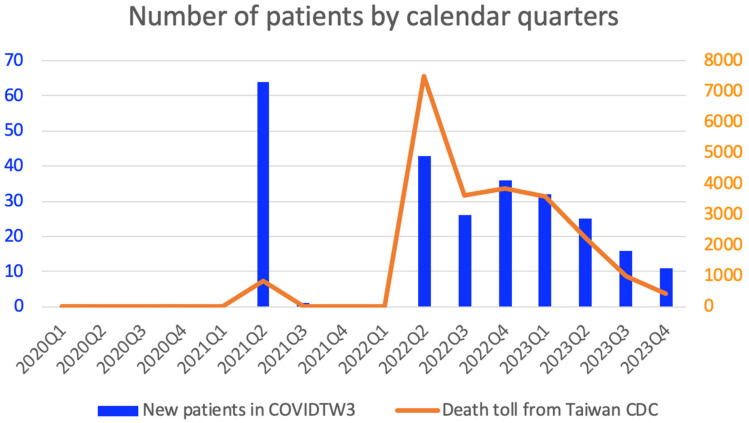
New COVID-19-related ARDS patients in the COVIDTW3 study and the death toll from the Taiwan Centers for Disease Control by calendar quarter. The blue bars represent the number of new patients in the COVIDTW3 study, which displays two peaks: the first peak indicates 64 new patients in the second quarter of 2021 (2021Q2), while the second peak indicates 43 new patients in the second quarter of 2022 (2022Q2). The orange curve indicates the number of deaths reported by the Taiwan CDC, which also shows two peaks: the first peak reflects 826 deaths in 2021Q2, and the second peak reflects 7488 deaths in 2022Q2.

Figure 1 illustrates the patient enrollment flow diagram for the COVIDTW3 study, which enrolled 254 patients with COVID-19-related ARDS. Among the 65 patients infected with the Alpha variant, all were unvaccinated. In contrast, of the 189 patients infected with the Omicron variant, 97 (51%) were vaccinated. The overall mortality rate for the study was 40%, with mortality rates of 45% for the Alpha variant and 38% for the Omicron variant. Figure 2 compares the new patient data from the COVIDTW3 study with the death toll reported by the Taiwan CDC across calendar quarters. Two significant epidemic waves were observed from 2021 to 2023. Generally, the trends in new patient cases in the COVIDTW3 study paralleled the death toll reported by the Taiwan CDC. However, a higher proportion of patients were enrolled during the first wave, which predominantly affected Northern Taiwan, with Taipei Tzu Chi Hospital serving as a key medical facility for COVID-19 treatment. The median age of the cohort was 75 years (IQR, 65–84; range, 27–99). Among these patients, 33 (13%) were younger than 60 years, while 221 (87%) were aged 60 years or older, consistent with a predominantly elderly study population.

Table 1 outlines the clinical characteristics of survivors compared to deceased patients. The deceased patients were generally older and had a lower BMI. In terms of comorbidities, they exhibited higher rates of malignancies but lower rates of tracheostomy prior to admission and mental disorders. On day 1, the SOFA scores indicated that deceased patients had higher total scores, reduced platelet counts, lower blood pressure, and impaired renal function. ABG parameters on day 1 revealed that deceased patients had decreased pH and bicarbonate levels. Additionally, there was a higher proportion of cisatracurium usage among deceased patients in the treatment of COVID-19.

To predict survival on day 1, we excluded mortality risk factors that arise post-intubation. Consequently, cisatracurium was omitted, as it is primarily used after intubation to facilitate patient–ventilator synchrony. Therefore, we included age, BMI, vaccination status, variant of concern, history of malignancy, tracheostomy prior to admission, mental disorders, the SOFA score, and serum bicarbonate value in our multivariate Cox proportional hazards regression analysis (Table 2). Ultimately, we found that older age, higher SOFA score, lower BMI, lower serum bicarbonate level, and infection with the Alpha variant were associated with an increased risk of mortality. The optimal cut-off values for these mortality risk factors were determined to be age ≥ 77 years, BMI ≤ 26.6 kg/m^2^, SOFA score ≥ 9 points, and serum bicarbonate ≤ 22.5 mEq/L (Supplementary Table S1).

**Figure 3:**
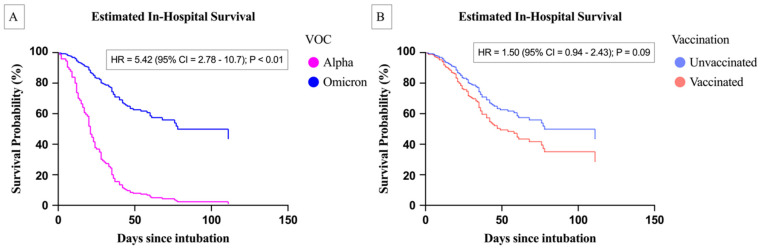
Estimated in-hospital survival based on variant of concern (**A**) and vaccination status (**B**) as determined by Cox proportional hazards regression. (**A**) The Alpha variant demonstrated a significantly higher mortality risk compared to the Omicron variant (hazard ratio = 5.42, 95% confidence interval: 2.78–10.7; *p* < 0.01). (**B**) Vaccinated patients exhibited a higher mortality risk than unvaccinated patients; however, this result did not reach statistical significance (hazard ratio = 1.5, 95% confidence interval: 0.94–2.43; *p* = 0.09).

Figure 3A presents the estimated in-hospital survival rates for the Alpha and Omicron variants, as determined by the Cox proportional hazards model. The Alpha variant exhibited a significantly higher mortality risk compared to the Omicron variant, with a hazard ratio of 5.42 (95% confidence interval: 2.78–10.7; *p* < 0.01). Figure 3B shows that vaccinated patients had a greater mortality risk than unvaccinated individuals; however, this finding did not reach statistical significance (hazard ratio: 1.5; 95% confidence interval: 0.94–2.43; *p* = 0.09). Table 3 outlines the secondary outcomes. Patients who died had shorter hospital stays and higher rates of septic shock, septicemia, fungemia, in-hospital cardiac arrest, and acute kidney injury. Supplementary Figure S1 illustrates the restricted cubic spline model used to analyze the non-linear relationship between BMI and mortality. We selected three spline knots at 18.5 kg/m^2^, 25 kg/m^2^, and 30 kg/m^2^, using a BMI of 18.5 kg/m^2^ as the reference point. The restricted cubic spline curve was divided into four segments by these three knots. The *p*-values for non-linearity across these segments were 0.55, 0.17, 0.47, and 0.77, respectively. The results did not indicate a significant non-linear association with BMI.

Groups 1, 2, and 3 included 35, 157, and 62 patients, respectively. After adjusting for age, BMI, VOC, SOFA score, and serum bicarbonate levels using the Cox proportional hazards model, no statistically significant differences in vaccine effectiveness were observed between the three groups. Specifically, the Bonferroni-adjusted *p*-values for the pairwise comparisons were *p* = 0.34 for Group 1 vs. Group 2, *p* = 0.99 for Group 1 vs. Group 3, and *p* = 0.29 for Group 2 vs. Group 3.

## 4. Discussion

This study specifically targeted intubated patients with COVID-19-related ARDS. The findings indicated that vaccination conferred no survival advantage, and the Alpha variant was associated with greater severity compared to the Omicron variant. Additionally, factors such as older age, higher SOFA score, lower BMI, and reduced serum bicarbonate levels were correlated with an increased risk of mortality.

The COVIDTW3 study concluded that prior vaccination did not improve the survival rate among intubated patients with COVID-19. By focusing exclusively on patients who required intubation, our study population is inherently susceptible to collider bias. The primary clinical value of vaccination lies in its profound effectiveness at the “gatekeeping” stage—significantly reducing the risk of progression from initial infection to critical illness and the need for mechanical ventilation. However, the effect of vaccination on survival in intubated patients with ARDS remains inconsistent in the current literature [9,13]. Variability in vaccine efficacy may arise from differences in VOC, types of vaccine, available treatments, and limited sample sizes. A multicenter observational study conducted in Greece, which included 265 patients, indicated that full vaccination was associated with a reduced risk of mortality among intubated patients with ARDS [13]. In this Greek study, the Delta variant was predominant, and the vaccines administered were BNT162b2 and ChAdOx1 nCoV-19/AZD1222. Conversely, a separate observational study in Japan involving 377 intubated patients found no significant impact of full vaccination on survival [9]. This Japanese study included various variants, and the specific vaccine types were not disclosed. Full vaccination rates in both studies (10% in Greece [13] and 9% in Japan [9]) were notably lower than in the COVIDTW3 study (35%). Despite vaccination reducing the progression to severe illness, vaccinated patients requiring IMV necessitated a comparable intensity of treatment.

The COVIDTW3 study revealed an increased mortality risk for intubated patients infected with the Alpha variant compared to those infected with the Omicron variant. These findings contrast with previous studies [9,11,12]. An observational study conducted in Japan, which included 377 intubated patients across four variants, reported a lower overall mortality rate compared to the COVIDTW3 study (18% vs. 40%). The Omicron variant demonstrated a higher mortality rate than the Alpha variant (31% vs. 12%), contradicting the COVIDTW3 results (38% vs. 45%). After conducting multivariate logistic regression, the variant type did not emerge as a significant risk factor for in-hospital death [9]. Another study in Northern Taiwan involving 94 intubated patients indicated a higher mortality rate associated with the Omicron variant compared to the Alpha variant (47% vs. 25%) [11]. However, multivariate Cox regression analysis indicated that variant type did not significantly influence in-hospital survival. A Brazilian study involving 231 intensive care units assessed the severity of non-variant, Gamma/Delta, and Omicron variants [12]. This study indicated that the Omicron variant exhibited a reduced mortality rate among critically ill patients; however, the variant type did not significantly affect mortality among those requiring IMV [12]. COVIDTW3 findings indicated that intubated patients with the Alpha variant infection experienced a higher mortality rate than those with the Omicron strain. Conversely, other studies suggested that variant type did not markedly affect survival outcomes [9,11,12]. Factors such as vaccine availability, epidemic wave dynamics, healthcare system strain, and evolving clinical guidelines may explain these observed discrepancies.

Our investigation identified several mortality risk factors, including advanced age, elevated SOFA scores, and decreased serum bicarbonate levels, which align with previous studies [9,14,15,16]. Sakuramoto et al. reported that age and SOFA score were independent predictors of mortality in mechanically ventilated patients [9]. Notably, the median age of the 377 intubated patients in that study was 69 years, which, although lower than the median age of 75 years observed in our cohort, still reflects a predominantly elderly population. Furthermore, a Japanese nationwide study of 1555 intubated COVID-19 patients demonstrated that, relative to patients aged 60 years, each successive decade of age was associated with a substantially increased mortality risk [15]. Although older age is a well-recognized risk factor for respiratory failure requiring mechanical ventilation, our findings suggest that among patients who are already intubated, heightened vigilance for age-related complications remains essential.

Limited research has specifically examined the effect of serum bicarbonate levels on COVID-19 survival [14,16]. A retrospective two-center study in Japan identified a U-shaped relationship between serum bicarbonate levels and mortality risk [14]. Both high and low bicarbonate levels were associated with increased risks of respiratory failure and mortality compared to normal levels. A South African study demonstrated that low serum bicarbonate is associated with higher mortality, consistent with our findings [16]. In critically ill patients with respiratory failure, reduced bicarbonate may reflect several interrelated mechanisms. It can serve as a marker of metabolic acidosis resulting from tissue hypoperfusion and increased anaerobic metabolism, both hallmarks of severe illness. Additionally, low bicarbonate may indicate underlying renal dysfunction, given the kidney’s central role in acid-base homeostasis; renal impairment commonly coexists with critical illness and independently worsens prognosis. Finally, in the setting of hypercapnic respiratory failure, low bicarbonate may reflect either insufficient renal compensation or a superimposed metabolic acidosis, further compromising physiological reserve.

Our research indicated that lower BMI was associated with increased mortality risk among intubated patients. However, restricted cubic spline analysis did not reveal a significant non-linear relationship with BMI. Previous studies have indicated a non-linear association between BMI and adverse outcomes [19,20]. A prospective study involving approximately 6.9 million patients in England revealed a U-shaped relationship between BMI and COVID-19 mortality risk. This community-based research indicated that both underweight and obesity increased the risks of hospitalization and death [19]. A meta-analysis encompassing various patient groups also demonstrated a U-shaped association between BMI and COVID-19 mortality [20]. Additionally, being overweight (BMI = 25–29.9 kg/m^2^) may confer a protective effect compared to other BMI categories [20]. Large-scale studies involving a substantial number of patients are more likely to reveal a U-shaped relationship between BMI and mortality risk [19,20]. In contrast, a smaller-scale Japanese study involving 377 patients found that BMI was not a significant risk factor for mortality among intubated patients with COVID-19 [9]. Our study is limited by its small sample size, consisting of 254 patients receiving IMV. Our findings suggest that a lower BMI may be associated with an increased mortality risk in this cohort. Specifically, a lower BMI may serve as a surrogate for frailty, underlying malnutrition, or sarcopenia, reflecting a diminished physiological reserve. These patients are often more vulnerable to the profound catabolic stress of sepsis and the prolonged physical demands of mechanical ventilation. Consequently, larger-scale studies are warranted to further elucidate the impact of BMI and nutritional status on the outcomes of critically ill COVID-19 patients.

Our study has several limitations that warrant careful interpretation. First, certain clinical factors remained unmeasured due to data constraints. Regarding the assessment of healthcare capacity, we lacked granular data on the intubation-to-ICU admission interval (measured in hours) for patients intubated in the general ward or emergency department—a factor known to critically influence the prognosis of acute respiratory failure. Second, supportive care protocols and the clinical environment evolved substantially between the two waves. During the Omicron period, prone positioning and post-extubation high-flow nasal cannula therapy became standardized components of critical care management. Additionally, the de-escalation of personal protective equipment requirements during the Omicron surge reduced the physical burden on healthcare providers, potentially improving the efficiency and frequency of bedside interventions compared to the highly restrictive environment of the Alpha wave. We cannot fully exclude the possibility that these unmeasured temporal shifts in healthcare resource delivery and clinical experience contributed to the improved survival observed during the Omicron period. Consequently, further studies are necessary to disentangle the relative contributions of viral virulence versus advancements in clinical care.

A noteworthy methodological limitation pertains to the lack of individual-level viral genomic sequencing. Variant classification was inferred based on the dominant circulating strain during predefined calendar periods, which introduces potential misclassification bias, particularly during transitional periods when multiple variants may co-circulate. However, Taiwan’s strict zero-COVID policy, encompassing rigorous border control, centralized quarantine, and systematic genomic surveillance, minimized simultaneous variant co-circulation. Genomic studies confirmed Alpha variant predominance during the 2021 outbreak [3,4] and Omicron sublineage dominance throughout 2022–2023 [4,5], supporting the validity of period-based classification. Variant-specific hazard ratios, particularly the association between Alpha variant infection and increased mortality risk, should nonetheless be interpreted with caution. As the COVID-19 pandemic has subsided and routine genomic surveillance is no longer conducted, a prospective validation study is no longer feasible, further underscoring the value of this retrospective analysis as a reference framework for future pandemic preparedness and management of novel respiratory pathogens.

## 5. Conclusions

The overall mortality rate for intubated patients experiencing respiratory failure due to COVID-19 was 40%. The COVIDTW3 study identified several factors associated with an increased mortality risk, including older age, higher SOFA scores, Alpha variant infection, lower serum bicarbonate levels, and lower BMI. Notably, vaccination status did not serve as a prognostic indicator.

## Figures and Tables

**Table 1:** Demographics and baseline characteristics.

Characteristics	Total (*n* = 254)	Survivors (*n* = 153)	Death (*n* = 101)	*p*-Value
Age, median (IQR), years	75 (65–84)	72 (63–83)	79 (70–85)	<0.01
Sex (male), *n* (%)	152 (60%)	93 (61%)	59 (58%)	0.79
BMI, median (IQR), kg/m^2^	23.9 (20.4–27.6)	24.1 (20.8–28.7)	23.3 (20.1–26.2)	0.04
Variants of concern, *n* (%)				0.35
Alpha variant (B.1.1.7)	65 (26%)	36 (24%)	29 (29%)	
Omicron variant (B.1.1.529)	189 (74%)	117 (76%)	72 (71%)	
Vaccination status, *n* (%)				
At least one dose	97 (38%)	56 (37%)	41 (41%)	0.52
At least two doses	88 (35%)	50 (33%)	38 (38%)	0.42
Ever-smoker, *n* (%)	63 (25%)	35 (23%)	28 (28%)	0.45
Bedridden status, *n* (%)	32 (13%)	22 (14%)	10 (10%)	0.34
Education status, *n* (%) ^a^	63 (25%)	41 (27%)	22 (22%)	0.38
Marital status, *n* (%)	207 (82%)	122 (80%)	85 (84%)	0.41
Out-of-hospital cardiac arrest, *n* (%)	7 (3%)	3 (2%)	4 (4%)	0.44
Comorbidities, *n* (%)				
Any	214 (84%)	131 (86%)	83 (82%)	0.46
Malignancy	31 (12%)	13 (9%)	18 (18%)	0.03
Diabetes mellitus	116 (46%)	69 (45%)	47 (47%)	0.90
Hypertension	100 (39%)	65 (43%)	35 (35%)	0.24
Cerebrovascular disease	42 (17%)	23 (15%)	19 (19%)	0.49
Epilepsy	6 (2%)	6 (4%)	0 (0%)	0.08
Parkinsonism	19 (8%)	10 (7%)	9 (9%)	0.48
Ischemic heart disease	35 (14%)	24 (16%)	11 (11%)	0.28
Congestive heart failure	45 (18%)	24 (16%)	21 (21%)	0.30
Tracheostomy prior to admission	9 (3.5%)	9 (6%)	0 (0%)	0.01
Asthma	13 (5%)	8 (5%)	5 (5%)	0.99
COPD	25 (10%)	12 (8%)	13 (13%)	0.19
Liver cirrhosis	7 (3%)	4 (3%)	3 (3%)	0.99
End-stage renal disease	24 (9%)	18 (12%)	6 (6%)	0.13
Dementia	30 (12%)	21 (14%)	9 (9%)	0.32
Mental disorders ^b^	15 (6%)	13 (9%)	2 (2%)	0.03
Autoimmune diseases ^c^	3 (1%)	0 (0%)	3 (3%)	0.06
Neuromuscular diseases ^d^	4 (2%)	3 (2%)	1 (1%)	>0.99
Number of comorbidities	2 (1–3)	2 (1–3)	2 (1–3)	0.39
SOFA score on day 1, median (IQR)	7 (5–9)	6 (4–8)	8 (5–11)	<0.01
Respiration (PaO_2_/FiO_2_)	4 (3–4)	4 (3–4)	4 (3–4)	0.65
Platelets	0 (0–1)	0 (0–1)	0 (0–1)	0.03
Bilirubin	0 (0–0)	0 (0–0)	0 (0–0)	0.08
Blood pressure	0 (0–1)	0 (0–1)	0 (0–1)	<0.01
Glasgow coma score	1 (0–3)	1 (0–3)	1 (0–3)	0.58
Kidney	0.5 (0–2)	0 (0–1)	1 (0–2)	<0.01
ABG parameters on Day 1, median (IQR)				
PH	7.40 (7.29–7.44)	7.40 (7.33–7.45)	7.30 (7.25–7.42)	<0.01
PaO_2_, mmHg	74.8 (54.1–128.5)	77.7 (54.2–131.9)	71.3 (52.4–120.7)	0.51
PaCO_2_, mmHg	37.6 (32.1–48.9)	38.7 (32.8–45.2)	37.1 (31.2–52.7)	0.69
HCO_3_^−^, mEq/L	22.4 (19.4–25.6)	23.5 (20.5–26.4)	20.4 (18.1–24.1)	<0.01
FiO_2_, %	52 (40–60)	60 (49–68)	50 (32–60)	0.48
SpO_2_, %	94.8 (85.4–98.9)	95 (87.7–99.1)	93.6 (83–98.7)	0.12
PaO_2_/FiO_2_	92.6 (62–152.5)	94.7 (62.4–162.5)	90.3 (61.1–151.9)	0.84
Medications for COVID-19, *n* (%)				
Oral antiviral medications ^e^	27 (11%)	18 (12%)	9 (9%)	0.538
Systemic steroids	243 (96%)	146 (96%)	97 (96%)	>0.99
Anticoagulants	152 (60%)	87 (57%)	65 (64%)	0.24
Remdesivir	231 (91%)	142 (93%)	89 (88%)	0.26
Tocilizumab	126 (50%)	72 (47%)	54 (54%)	0.37
Cisatracurium	104 (41)	54 (35%)	50 (50%)	0.03

Abbreviations: IQR, interquartile range; BMI, body mass index; COPD, chronic obstructive pulmonary disease; SOFA, Sequential Organ Failure Assessment; ABG, arterial blood gas. The *p*-values < 0.05 are indicated in red. ^a^ Education status indicates a bachelor’s degree or higher. ^b^ Mental disorders include bipolar disorder, depression, and schizophrenia. ^c^ Autoimmune diseases include systemic lupus erythematosus, Sjögren’s syndrome, and rheumatoid arthritis. ^d^ Neuromuscular diseases include myasthenia gravis, muscular dystrophy, Kennedy’s disease, and neuronal intranuclear inclusion disease. ^e^ Oral antiviral medications include nirmatrelvir/ritonavir (Paxlovid) and molnupiravir (Lagevrio).

**Table 2:** Risk factors for in-hospital mortality: a multivariate Cox proportional hazards regression analysis.

Risk Factors	Hazard Ratio (95% CI)	*p*-Value
Age	1.02 (1.01–1.04)	0.04
BMI	0.95 (0.91–0.99)	0.04
Vaccination status (At least one dose)	1.50 (0.94–2.43)	0.09
Variant of concern (Reference: Omicron)	5.42 (2.78–10.7)	<0.01
Malignancy	1.48 (0.85–2.45)	0.15
Tracheostomy prior to admission	<0.01 (inestimable) ^a^	0.99
Mental disorders	0.32 (0.05–1.07)	0.12
SOFA scores	1.11 (1.02–1.20)	0.01
Serum bicarbonate (HCO3^−^)	0.94 (0.90–0.98)	<0.01

Abbreviations: CI, confidence interval; BMI, body mass index; SOFA, Sequential Organ Failure Assessment. *p*-values < 0.05 are indicated in red. ^a^ The 95% confidence interval is not estimable due to complete data separation.

**Table 3:** Secondary outcomes.

Secondary Outcomes	Total (*n* = 254)	Survivors (*n* = 153)	Death (*n* = 101)	*p*-Value
Length of hospital stay, median (IQR), days	31 (20–49)	35 (23–60)	24 (15–37)	<0.01
Duration of IMV, median (IQR), days	15 (9–27)	14 (9–25)	17 (9–30)	0.21
Septic shock, *n* (%)	83 (33%)	21 (14%)	62 (61%)	<0.01
Septicemia, *n* (%) ^a^	30 (12%)	13 (9%)	17 (17%)	0.04
Bacteremia	24 (9%)	14 (9%)	11 (11%)	0.52
Fungemia	8 (3%)	0 (0%)	8 (8%)	<0.01
CMV infection, *n* (%) ^b^	11 (4%)	6 (4%)	5 (5%)	0.76
Aspergillus infection, *n* (%) ^c^	14 (6%)	8 (5%)	6 (6%)	0.81
ECMO, *n* (%)	5 (2%)	3 (2%)	2 (2%)	>0.99
In-hospital cardiac arrest, *n* (%)	8 (3%)	0 (0%)	8 (8%)	<0.01
In-hospital stroke, *n* (%)	7 (3%)	4 (3%)	3 (3%)	>0.99
In-hospital seizure, *n* (%)	5 (2%)	2 (1%)	3 (3%)	0.39
Acute kidney injury, *n* (%) ^d^	66 (26%)	19 (12%)	47 (47%)	<0.01
Empyema, *n* (%)	4 (2%)	2 (1%)	2 (2%)	0.65
Pneumothorax, *n* (%)	5 (2%)	2 (1%)	3 (3%)	0.39
Post-tracheostomy status, *n* (%) ^e^	11 (4%)	6 (4%)	5 (5%)	0.76

Abbreviations: IQR, interquartile range; IMV, invasive mechanical ventilation; CMV, cytomegalovirus; ECMO, extracorporeal membrane oxygenation. The *p*-values < 0.05 are indicated in red. ^a^ Septicemia refers to both bacteremia and fungemia. ^b^ CMV infection is defined as a serum CMV viral load of ≥500 IU/mL or a positive CMV-IgM test. ^c^ Aspergillus infection is characterized by a positive sputum culture or an Aspergillus galactomannan antigen level of ≥0.5 (optical density index) in serum or bronchoalveolar lavage fluid. ^d^ Acute kidney injury is defined as an increase in serum creatinine to ≥1.5 times baseline during hospitalization. ^e^ Post-tracheostomy status indicates that the patient had undergone tracheostomy surgery after admission.

## Data Availability

The original contributions presented in this study are included in the article/Supplementary Material. Further inquiries can be directed to the corresponding author.

## Supplementary Materials

The following supporting information can be downloaded at: https://www.mdpi.com/article/10.3390/biomedicines14040756/s1, Figure S1: Restricted cubic spline model for the non-linear relationship between body mass index (BMI) and mortality. Table S1: Optimal cut-off values for mortality risk factors determined by the Youden index.

## Abbreviations

SARS-CoV-2	severe acute respiratory syndrome coronavirus 2
CDC	Centers for Disease Control
VOC	variant of concern
ARDS	acute respiratory distress syndrome
SOFA	Sequential Organ Failure Assessment
IMV	invasive mechanical ventilation
ABG	arterial blood gas
CMV	cytomegalovirus
ECMO	extracorporeal membrane oxygenation
IQR	interquartile range
ROC	receiver operating characteristic
ICU	intensive care unit
