# Supplementary material for: The COVIDTW3 Study: Impact of Variants of Concern and Vaccination on Mortality in Intubated Patients with COVID-19-Related Respiratory Failure from 2021 to 2023
Source: Biomedicines. 2026 Mar 26;14(4):756. doi: 10.3390/biomedicines14040756 (PMC13113807; doi:10.3390/biomedicines14040756)
Supplement: Supplementary file 1 [file biomedicines-14-00756-s001.zip › biomedicines-4163952-supplementary.pdf]

Supplementary Figure S1. Restricted cubic spline model for the non-linear relationship between body mass index (BMI) and mortality.

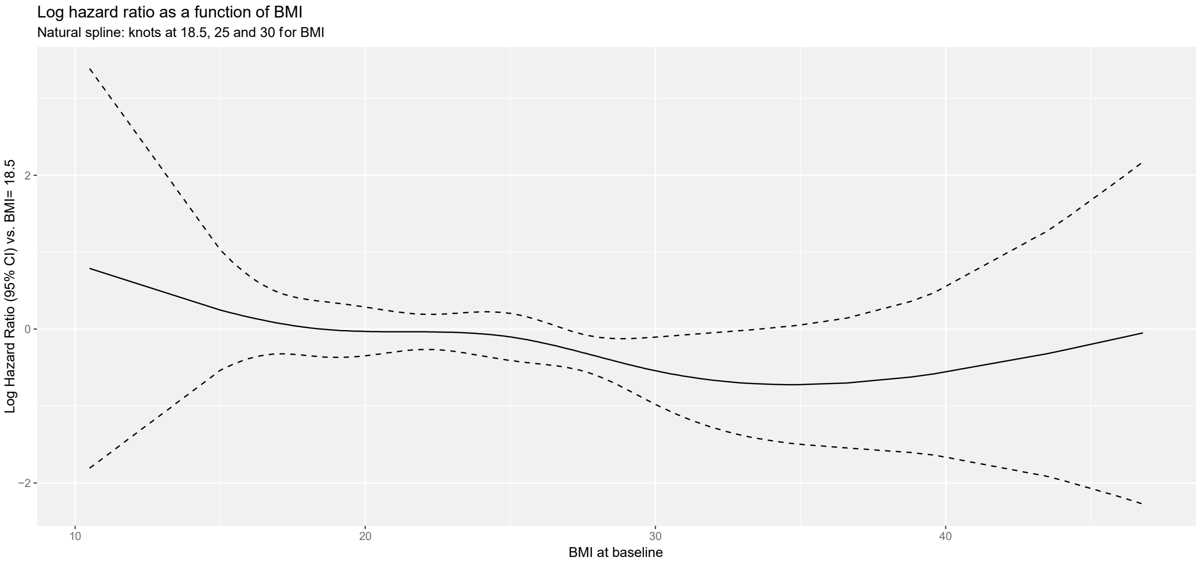

The x-axis represents body mass index (BMI) in kg/m², and the y-axis displays the log hazard ratio with 95% confidence intervals, using a BMI of 18.5 kg/m² as the reference point. Three spline knots were placed at 18.5, 25, and 30 kg/m², dividing the curve into four segments. P values for non-linearity across these segments were 0.55, 0.17, 0.47, and 0.77, respectively, indicating no significant non-linear association between BMI and mortality.

Supplementary Table S1. Optimal cut-off values for mortality risk factors determined by the Youden index.

Risk factors	Area under ROC curve (95% CI)	P value	Cut-off value
Age, years	0.62 (0.55 – 0.69)	< 0.01	77
BMI, kg/m^2	0.58 (0.51 – 0.65)	0.04	26.6
SOFA score	0.62 (0.54 – 0.69)	< 0.01	9
Serum bicarbonate, mEq/L	0.66 (0.59 – 0.73)	< 0.01	22.5

Abbreviations: CI, confidence interval; ROC, receiver operating characteristic; BMI, body mass index; SOFA, Sequential Organ Failure Assessment.
